# Prognostic models for complete recovery in ischemic stroke: a systematic review and meta-analysis

**DOI:** 10.1186/s12883-018-1032-5

**Published:** 2018-03-09

**Authors:** Nampet Jampathong, Malinee Laopaiboon, Siwanon Rattanakanokchai, Porjai Pattanittum

**Affiliations:** Department of Epidemiology and Biostatistics, Faculty of Public Health, Khon Kaen University, 123 Mittraphap Road, Nai-Muang, Muang District, Khon Kaen, 40002 Thailand

**Keywords:** Stroke, Cerebral ischemia, Prognosis, Ischemic stroke, Prognostic model, Systematic review

## Abstract

**Background:**

Prognostic models have been increasingly developed to predict complete recovery in ischemic stroke. However, questions arise about the performance characteristics of these models. The aim of this study was to systematically review and synthesize performance of existing prognostic models for complete recovery in ischemic stroke.

**Methods:**

We searched journal publications indexed in PUBMED, SCOPUS, CENTRAL, ISI Web of Science and OVID MEDLINE from inception until 4 December, 2017, for studies designed to develop and/or validate prognostic models for predicting complete recovery in ischemic stroke patients. Two reviewers independently examined titles and abstracts, and assessed whether each study met the pre-defined inclusion criteria and also independently extracted information about model development and performance. We evaluated validation of the models by medians of the area under the receiver operating characteristic curve (AUC) or c-statistic and calibration performance. We used a random-effects meta-analysis to pool AUC values.

**Results:**

We included 10 studies with 23 models developed from elderly patients with a moderately severe ischemic stroke, mainly in three high income countries. Sample sizes for each study ranged from 75 to 4441. Logistic regression was the only analytical strategy used to develop the models. The number of various predictors varied from one to 11. Internal validation was performed in 12 models with a median AUC of 0.80 (95% CI 0.73 to 0.84). One model reported good calibration. Nine models reported external validation with a median AUC of 0.80 (95% CI 0.76 to 0.82). Four models showed good discrimination and calibration on external validation. The pooled AUC of the two validation models of the same developed model was 0.78 (95% CI 0.71 to 0.85).

**Conclusions:**

The performance of the 23 models found in the systematic review varied from fair to good in terms of internal and external validation. Further models should be developed with internal and external validation in low and middle income countries.

**Electronic supplementary material:**

The online version of this article (10.1186/s12883-018-1032-5) contains supplementary material, which is available to authorized users.

## Background

Globally, stroke is the second leading cause of death following ischemic heart disease and the third leading cause of disability [1, 2]. In 2013, 6.5 million deaths from stroke (51% died from ischemic stroke), 113 million disability-adjusted life years were lost because of stroke (58% due to ischemic stroke) and 10.3 million of people with new strokes (67% were ischemic stroke) [1]. In 2015, prevalence of stroke was 42.4 million people, which included ischemic stroke for 24.9 million. There were 6.3 million stroke deaths worldwide, and 3.0 million individuals died of ischemic stroke [2].

Minimizing the time to treatment for stroke is the important key to improving chances of an excellent outcome (time lost is brain lost) [3]. It is also important to be able to predict the outcomes of diseases or treatments. Most physicians use their own clinical experience in predicting their patients’ outcomes for making decisions in patient care management. The accuracy of these informal predictions is unclear. Care management might be improved if the physicians combined their clinical forecasts with the formal predictions provided by statistical models. This may be more accurate than relying simply on clinical experience. Prognostic models are statistical tools to assist physicians in making decisions which may affect their patients’ outcomes [4].

Accurate prognostic models of the functional outcome of a complete recovery in patients after ischemic stroke could be beneficial to neurological care practices for a number of reasons. Firstly, the information of developed prognostic model could be used to select appropriate treatments and action plans in individual patient management, including patient counseling. Secondly, they could be used to improve rehabilitation and discharge planning. Lastly, in light of a weakening economy, prognostic models could be used to make the best clinical choices for patients with regard to specific clinical scenarios which may reduce health care costs [5].

To date, several studies have developed prognostic models to predict functional outcomes after ischemic stroke, and each model has different strengths and weaknesses. Since models do not always work well in practice, it is recommended that, before a prognostic model is used in clinical practice, the performance of the model should be properly evaluated. This process is known as model validation and involves an assessment of calibration (the agreement between the observed and predicted outcomes) and discrimination (the model’s ability to discriminate between those patients who are likely or unlikely to experience a particular prognostic event). A poor calibration usually reflects over-fitting of the model in the development sample. At least the models should be determined the internal validity (for example, using ‘bootstrap sampling’) to assess validity for the setting where the development data originated from. Another aspect is the external validity (using patient data not used for the development model) to assess generalizability [6, 7].

There may be danger in moving too quickly to use these models without appropriate validation and understanding of their limitations. The purpose of this study was to systematically review and synthesize performance of existing prognostic models which have been used to predict the probability of complete recovery in ischemic stroke and to investigate their quality.

## Methods

### Selection criteria

We included studies predicting the outcome of complete recovery after ischemic stroke and in which complete recovery was assessed by scores on at least one of the following instruments: the Barthel Index (BI) ≥ 95/100 or 19/20, the Glasgow Outcome Scale (GOS) score = 1, the Oxford Handicap Scale (OHS) score ≤ 2, and the Modified Rankin Scale (mRS) score ≤ 1. A further criterion was that the studies reported model performance by the use of the concordance statistic, area under the receiver operating characteristic curve (AUC) or calibration performance. There were no restrictions on timing of the outcome evaluation, age of the patients, or type/severity of ischemic stroke.

### Search strategy

We searched PUBMED, SCOPUS, CENTRAL, ISI Web of Science and OVID MEDLINE for prognostic models published from inception until 4 December, 2017, using the search terms listed in the Additional file 1 without restrictions on publication language. We also reviewed the reference lists of relevant studies.

### Study selection and data extraction

Study titles and abstracts were independently screened and selected by two reviewers (NJ and SR) using the specified criteria. If a decision could not be made based on the abstracts, we then considered their full texts. Disagreement was resolved through discussion with a third reviewer (ML). We extracted the performance measures (concordance statistics, AUCs and performance calibrations) of both types of prediction model: development models and validation models. We also extracted study characteristics: author(s), publication year, setting, study design, definition of outcome, number of subjects, number of outcome events, age, ischemic stroke severity and duration of follow-up.

### Quality assessment

We assessed the study quality based on an adaptation of the tool developed by D’Amico et al. [8]. We showed how each study performed according to each of various major methodological requirements for prognosis research studies. The assessment items were as follows:

- Did the prognostic study use a cohort design?
- Were the predictors clearly defined and details provided of how they were measured?
- Were the missing data handled appropriately with statistical imputation?
- Was some form of stepwise analysis used for selecting predictors in a multivariable analysis?
- Was the sample size adequate as defined by an events-per-variable ratio of 10 or more?
- Was the final model validated on the patients who were used to generate the model (internal validation)?
- Was the final model validated on the patients who were not used to generate the model (external validation)?

### Statistical analysis

We qualitatively synthesized model performances because each separate model had a different combination of predictor variables. We used frequencies and medians with 95% confidence intervals to describe the model performance which included its calibration (how closely predicted values agree with the observed values) and discrimination (the model’s ability to discriminate between patients developing and not developing an outcome event, e.g., complete recovery cases and non-complete recovery cases among ischemic patients). The assessment of calibration was performed using either the Hosmer-Lemshow chi-square test or a calibration curve. The assessment of discrimination was conducted using either the AUC or the concordance statistic (C-statistic) along with a 95% CI. The discrimination of each model was evaluated in accordance with the suggestions by Hosmer and Lemeshow: excellent (AUC ≥ 0.90), good (AUC ≥ 0.80 and ˂ 0.90), fair (AUC ≥ 0.70 and ˂ 0.80), and poor (AUC ˂ 0.70). Calibration was judged as good when a calibration curve closely resembled the line representing perfect calibration (the pre-specified acceptable absolute mean error for the calibration curve was ˂ 0.4) or when the Hosmer-Lemshow chi-square test was non-significant [9, 10]. We estimated the 95% CIs for AUCs using Hanley’s method for a study which presented only AUCs. The estimation required three quantities: total sample size, number of events and an AUC [11]. If two or more models assessed discrimination performance in terms of validation, we performed a random-effects inverse-variance meta-analysis using Stata version 10.1 [12].

## Results

**Fig. 1:**
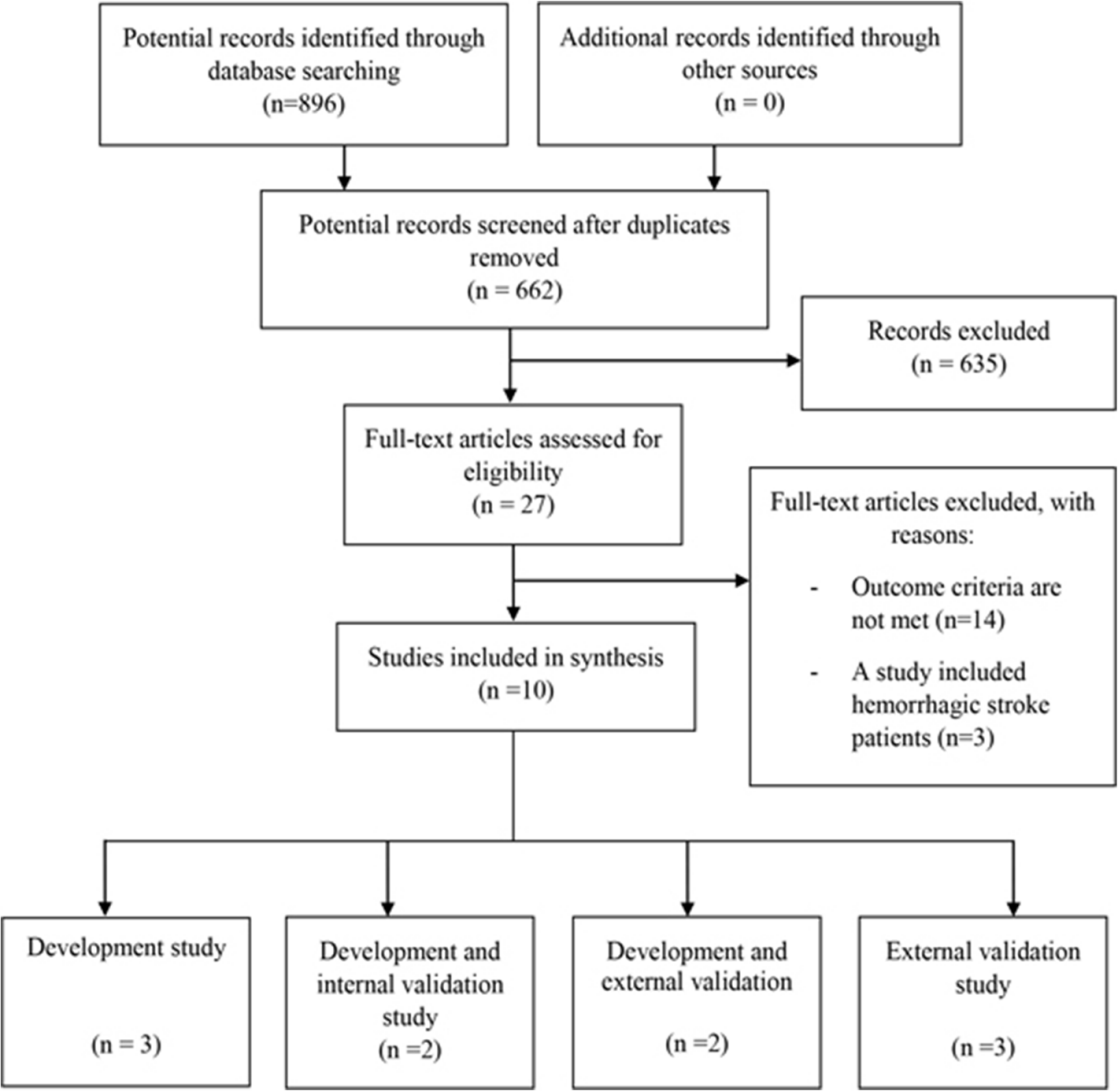
Study flow diagram

A total of 896 articles were found by searching the electronic databases, and 10 studies were eligible [13–22]. Seven studies were development studies; while three were validation studies (see Fig. 1). Twenty three different models were identified.

### Patient characteristics of included models

All included models were developed from elderly patients in high income countries: five studies from the United States of America [13–16, 22], three from Germany [17–19] and one from the Netherlands [21]. One study [20] did not report study setting but used data from the Virtual International Stroke Trials Archive (VISTA) which included patients from many different countries.

Twenty models were developed from patients with a moderately severe ischemic stroke based on the NIHSS score [23], but for three models ischemic stroke severity was not reported. The sample sizes from which the models were developed ranged from 75 to 4441, the complete recovery cases ranged from 33 to 1970 and were measured at around 90 days after ischemic stroke diagnosed in six studies [13–16, 20, 22], at 100 days in three studies [17–19], and at 365 days in one study [21]. Table 1 presents the characteristics of the models.

**Table 1:** Characteristics of prognostic models

First author (year)	Setting	Study period	Study design	Model No.	Type of model	Definition of outcome	Participants				No. of complete recovery	Events per variable	Duration of follow-up
							n	Age	Male	NIHSS score			
Johnston (2000)	America	May 1993 - Dec 1994	Cohort	1	Internal	BI ≥ 95	222	68.7^b^	–	10 (5, 15)^a^	125	125	90 days
				2								21	
				3								18	
				4		GOS = 1	222	68.7^b^	–	10 (5, 15)^a^	108	108	
				5								18	
				6								16	
Johnston (2003)	America	–	Cohort	3	External	BI ≥ 95	199	67 (59,74)^a^	59%	15(10, 20)^a^	78	12	90 days
				6		GOS = 1					62	9	
Johnston (2002)	America	May 1993 - Dec 1994	Cohort	7	Internal	BI ≥ 95	206	69 (12)^b^	–	10 (5, 16)^a^	92	92	90 days
				8								92	
				9								46	
				10		GOS = 1	206	69 (12)^b^	–	10 (5, 16)^a^	99	99	
				11								99	
				12								50	
Johnston (2007)	America	–	Cohort	13	Development	BI ≥ 95	382	69 (58, 77)^a^	53.9%	14 (10, 18)^a^	123	25	90 days
		May 2000 & Aug 2005			External		266	70 (58, 78)^a^	53.0%	5 (3, 10)^a^	167	34	
		–		14	Development		382	69 (58, 77)^a^	53.9%	14 (10, 18)^a^	123	16	
		May 2000 & Aug 2005			External		266	70 (58, 78)^a^	53.0%	5 (3, 10)^a^	167	21	
		–		15	Development	mRS ≤ 1	382	69 (58, 77)^a^	53.9%	14 (10, 18)^a^	75	15	
		May 2000 & Aug 2005			External		266	70 (58, 78)^a^	53.0%	5 (3, 10)^a^	148	30	
		–		16	Development		382	69(58, 77)^a^	53.9%	14 (10, 18)^a^	75	10	
		May 2000 & Aug 2005		16	External	mRS ≤ 1	266	70 (58, 78)^a^	53.0%	5 (3, 10)^a^	148	19	90 days
Weimar (2002)	Germany	1998 - 1999	Cohort	17	Development	BI ≥ 95	1743	68.1 (12.7)^b^	59.2%	6.9 (6.2)^b^	1021	102	100 days
German Stroke Study Collaboration (2004)	Germany	Feb 2001 - Mar 2002	Cohort		External	BI ≥ 95	1470	67.9 (12.4)^b^	57.3%	6.4 (6.0)^b^	831	76	100 days
Weimar (2004)	Germany	–	Cohort	18	Development	BI ≥ 95	1079	67.0 (12.3)^b^	60.5%	–	644	322	100 days
		Feb 2001 - Mar 2002	Cohort		External	BI ≥ 95	1307	68.2 (12.5)^b^	56.5%	7.6 (6.9)^b^	722	361	100 days
Konig (2008)	–	–	Cohort		External	BI ≥ 95	4441	68.8 (12.3)^b^	55.8%	13.4 (6.5)^b^	1970	985	90 days
Schiemanck (2006)	Netherland	1999 - 2001	Cohort	19	Development	BI ≥ 19	75	63 (15)^b^	47%	11 (6)^b^	33	17	365 days
				20								7	
Patti (2016)	America	2013 - 2014	Cohort	21	Development	mRS ≤ 1	414	70 (50, 69)^a^	50%	5 (2, 13)^a^	230	77	90 days
				22			204	–	–	–	–	–	
				23			210	–	–	–	–	–	

^a^Median (25th–75th percentile); ^b^ Mean (SD); − indicates not stated

### Model predictors

A total of 24 different variables were included in the 23 models. The number of variables included in each model ranged from 1 to 11. The National Institutes of Health Stroke Scale (NIHSS) was the most common predictor (70.8%) followed by age (62.5%) and infarct volume (50.0%). Table 2 presents details of predictor variables.

**Table 2:** Predictors included in final model

No. of model	1	2	3	4	5	6	7	8	9	10	11	12	13	14	15	16	17	18	19	20	21	22	23	Total (%)	
Predictors included																									
NIHSS score		*	*		*	*	*		*	*		*	*	*	*	*	*	*			*	*	*	17	(70.8)
Age		*	*		*	*							*	*	*	*	*	*	*	*	*	*	*	15	(62.5)
Infarct volume	*		*	*		*		*	*		*	*								*				12	(50.0)
Infarct volume < 29.5 mL																					*				
Infarct volume < 31.2 mL																						*			
Infarct volume < 25.5 mL																							*		
History of diabetes mellitus		*	*		*	*							*	*	*	*	*							9	(37.5)
History of stroke		*	*		*	*											*				*	*	*	8	(33.3)
Prestroke disability		*	*		*	*							*	*	*	*								8	(33.3)
Small-vessel stroke		*	*		*	*																		4	(16.7)
Tissue-type plasminogen activator (t-PA use)													*	*	*	*								4	(16.7)
Preadmission modified Rankin scale																	*				*	*	*	4	(16.7)
Sex																	*				*	*	*	4	(16.7)
Atrial fibrillation																					*	*	*	3	(12.5)
Congestive heart failure																					*	*	*	3	(12.5)
Antiplatelet use																					*	*	*	3	(12.5)
Diffusion-weighted imaging lesion volume (DWI)														*		*								2	(8.3)
Time to DWI scan														*		*								2	(8.3)
Time by DWI interaction														*		*								2	(8.3)
Barthel index																			*	*				2	(8.3)
Neurological complications																	*							1	(4.2)
Fever > 38 °C																	*							1	(4.2)
Lenticulostriate arteries infarction																	*							1	(4.2)
Right arm weakness																	*							1	(4.2)
Left arm weakness																	*							1	(4.2)
Days to poststroke MRI scan																				*				1	(4.2)
Hemisphere (left/right)																				*				1	(4.2)
Total	1	6	7	1	6	7	1	1	2	1	1	2	5	8	5	8	11	2	2	5	9	9	9		

*indicates the predictor included in the final model

### Quality of prognostic models

All 10 studies used a cohort design. Details about the measurement of predictors were presented for 13 of the 23 models (56.5%). All of the 10 studies handled missing data by excluding subjects from the analyses. The full model approach (all the candidate predictors included in the multivariable analysis) was the most common method used (69.6%) for selection of predictors by multivariable modeling [13, 14, 16]. Two studies [13, 14] reported 12 development models (model No.1-12) but provided no information on their discrimination performances. However, they did report their discrimination performances on internal validation, but for only one model (model No. 9) was calibration performance reported in the internal validation. Five studies [15, 16, 18–20] reported external validation in nine models (model No.3, 6 and 13-18). Calibration performances were reported for six of these nine models, and four of them (model No. 3, 6, 13 and 15) showed good calibration. There was only one study which provided a 95% confidence interval for the AUC [21]; those of the other nine studies [13–20, 22] were estimated using Hanley’s method (see Table 4). There were only two models (model No.3 and No.6) which were validated both internally and externally. Table 3 presents details of quality of prognostic models.

**Table 3:** Quality assessment of prognostic models

Assessment items	All models (*n* = 23)
Study design	
Cohort study	23 (100%)
Variables	
Description of measurement of predictors	
Yes	13 (56.5%)
No	10 (43.5%)
Loss to follow-up	
< 10%	10 (43.5%)
≥ 10%	13 (56.5%)
Analysis	
More than 10 events per variable	
Yes	22 (95.7%)
No	1 (4.3%)
Method for selection of predictors during multivariable modeling	
Forward Selection	2 (8.7%)
Backward Elimination	3 (13.0%)
Stepwise selection	0
Full model approach	16 (69.6%)
Unknown	2 (8.7%)
Handling of missing data	
Estimated statistically	0
Excluded	23 (100%)
Model performance	
Internal validity	
Performance reported AUC (Discrimination)	
Yes	12 (52.2%)
95% CI presented	0
No	11 (47.8%)
Calibration	
Yes	1 (4.3)
No	22 (95.7%)
External validity	
Performance reported AUC (Discrimination)	
Yes	8 (34.9%)
95% CI presented	2 out of 8 (25.0%)
No	15 (65.1%)
Calibration	
Yes	6 (26.1%)
No	17 (73.9%)

### Model performances

**Fig. 2:**
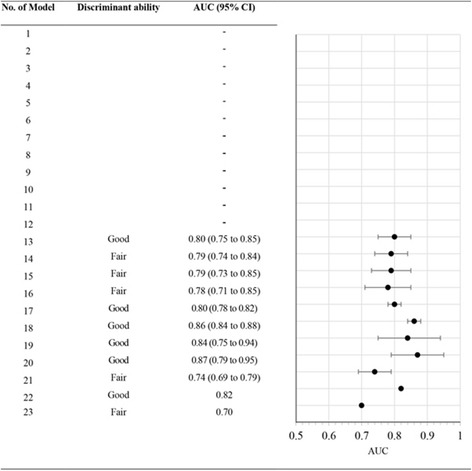
Discrimination performance in development models

There were 11 development models which reported AUC values. The median AUC was 0.80 (95% CI 0.77 to 0.85) (see Table 4 and Fig. 2).

**Table 4:** Model performances

No. of model	Calibration		Discrimination; AUC (95%CI)		
	Internal validation	External validation	Development model	Internal validation	External validation
1	–	–	–	0.73 (0.66 to 0.80)	–
2	–	–	–	0.80 (0.74 to 0.86)	–
3	–	closely resembling perfect calibration	–	0.84 (0.79 to 0.89)	0.83 (0.77 to 0.89)
4	–	–	–	0.74 (0.67 to 0.81)	–
5	–	–	–	0.79 (0.73 to 0.85)	–
6	–	closely resembling perfect calibration	–	0.84 (0.79 to 0.89)	0.81 (0.75 to 0.87)
7	–	–	–	0.87 (0.82 to 0.92)	–
8	–	–	–	0.70 (0.63 to 0.77)	–
9	closely resembling perfect calibration, mean absolute error = 0.01	–	–	0.87 (0.82 to 0.92)	–
10	–	–	–	0.89 (0.84 to 0.94)	–
11	–	–	–	0.72 (0.65 to 0.79)	–
12	–	–	–	0.89 (0.84 to 0.94)	–
13	–	closely resembling perfect calibration, mean absolute error = 0.33	0.80 (0.75 to 0.85)	–	0.82 (0.76 to 0.88)
14	–	mean absolute errors = 0.4, less well calibrated	0.79 (0.74 to 0.84)	–	0.80 (0.73 to 0.87)
15	–	closely resembling perfect calibration, mean absolute error = 0.37	0.79 (0.73 to 0.85)	–	0.80 (0.74 to 0.86)
16	–	mean absolute errors = 0.4, less well calibrated	0.78 (0.71 to 0.85)	–	0.76 (0.69 to 0.83)
17	–	–	0.80 (0.78 to 0.82)	–	0.78 (0.75 to 0.81)
18	–	–	0.86 (0.84 to 0.88)	–	0.74 (0.71 to 0.77)
					0.81 (0.80 to 0.82)
19	–	–	0.84 (0.75 to 0.94)	–	–
20	–	–	0.87 (0.79 to 0.95)	–	–
21	–	–	0.74 (0.69 to 0.79)	–	–
22	–	–	0.82	–	–
23	–	–	0.70	–	–
Median AUC (95% CI)			0.80 (0.77 to 0.85)	0.82 (0.73 to 0.87)	0.80 (0.76 to 0.82)

#### Internal validation

**Fig. 3:**
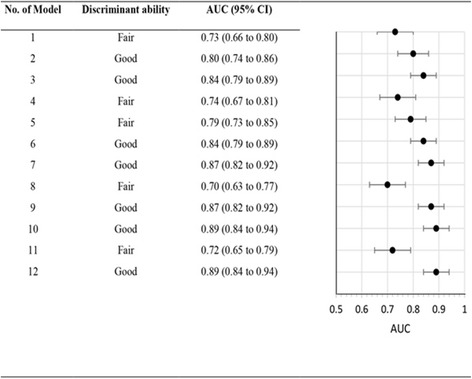
Discrimination performance in internal validation models

Model performance was evaluated using internal validation by bootstrapping methods in 12 models (model No.1-12). The models were validated in samples of 222 [13] and 206 [14] elderly patients with a moderately severe ischemic stroke. The number of events ranged from 92 to 125 (see Table 1). The median AUC for discrimination performance on internal validation was 0.82 (95% CI 0.73 to 0.87); there was only model No. 9 which was reported to have good calibration (see Table 4 and Fig. 3).

#### External validation

Two studies [16, 19] reported external validation in their five developed models (model No.13-16 and 18). Model No.18 was validated in two different samples in two studies [19, 20]; one study included patients from Germany [19] and another study included patients from many countries [20]. Three other studies [15, 18, 20] reported external validation of four pre-existing models (model No 3, 6, 17 and 18).

**Fig. 4:**
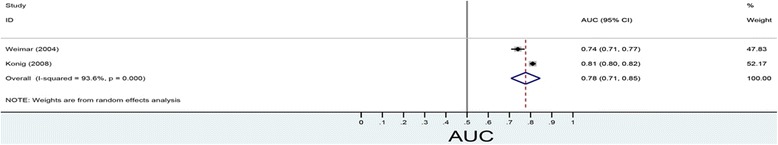
Meta-analysis of the areas under the receiver operating characteristic curve (AUC) for previous prognostic models

**Fig. 5:**
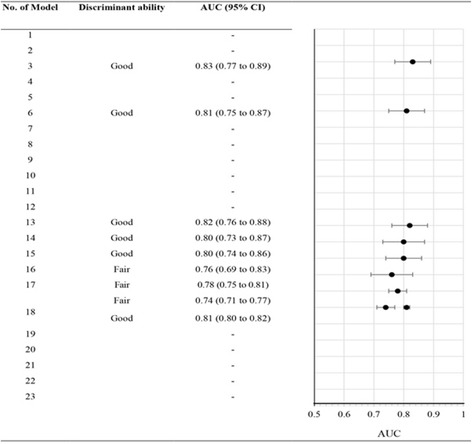
Discrimination performance in external validation models

On external validation discrimination and calibration performance were reported in six models (model No. 3, 6 and 13-16). Four of the six models had good discrimination with a median AUC of 0.81 (95% CI 0.80 to 0.83), and good calibration (model No. 3, 6, 13 and 15). Three other models (model No.17, model No.18 reported in two external populations) reported only discrimination performance. The pooled AUC value for model No. 18 was 0.78 (95% CI 0.71 to 0.85; two studies) (see Fig. 4). The median AUC of these nine validation models was 0.80 (95% CI 0.76 to 0.82) (see Table 4 and Fig. 5).

## Discussion

This systematic review identified 23 prognostic models from ten studies for complete recovery in ischemic stroke. None of these models provided complete information about the model performance which included both internal and external validation. While most prognostic models (18/23) were validated and half of the models (12/23) reported fair to good discrimination on internal validation, only one model showed good calibration. Nearly one third of the models (9/23) were externally validated, and reported fair to good discrimination performance, but only a quarter of the models (6/23) reported nearly perfect calibration. Only two models were validated both internally and externally but not in complete process of the model performance. There was only one model in which a meta-analysis could be performed, and the pooled AUC was fair.

The models were developed and validated in elderly patients mainly with a moderately severe stroke and mainly from high income countries. In addition, most of the developed models were not externally validated. These factors are likely to limit the application of the models to other populations and settings.

In our review, we conducted a systematic search of several electronic databases. All of the included studies used a cohort design. For half of all the identified models more than 10% of their subjects’ data was missing. The model performance analyses were handled by excluding the subjects with missing data. This strategy could lead to biased conclusions if the reasons for missing data were related to the important prognostic indicators or outcomes.

There are some issues related to the assessed quality of the prognostic models. Firstly, our search was performed up to 4 December, 2017. No attempt was made to search unpublished studies. Studies were selected and extracted independently by two reviewers. We did not assess publication bias by any statistical tests or funnel plot asymmetry due to insufficient data. However, we assessed and presented the quality of all the 10 selected studies for each important quality features listed in our methods section. Secondly, about 70% (16/23) of the models used the full model approach in predictor selection (all the candidate predictors included in the multivariable analysis) [13, 14, 16]. This approach could reduce the risk of predictor selection bias and over-fitting. However, this technique is difficult to apply if the number of events is limited [6]. In our review, all of the models with the full model approach to predictor selection had fulfilled the requirement of more than 10 patients with complete recovery per predictor. Finally, incomplete measures of the prognostic model performances were reported in all included models. The 95% confidence intervals of the estimated performance indices were rarely reported. The 95% confidence intervals for AUCs which we estimated using Hanley’s method may be slightly inaccurate, but this approach has been accepted in estimating the precision of AUCs when their standard errors are not reported. In addition, calibration performance was often ignored. Calibration is the important performance measure for application of the model in practice. A poor calibration reflects over-fitting of a model and can also be interpreted as reflecting a need for shrinkage of regression coefficients in a prognostic model [10].

To our knowledge, there are three previous systematic reviews of prognostic models in stroke, but their outcomes of interest were different from ours: for example, mortality in hemorrhagic stroke, recurrent stroke and survival outcome of stroke patients. Therefore, our results were not able to be compared directly to the results of previous reviews. However, while the discrimination performance of their prognostic models varied from poor to good, calibration performance was not considered. The first study was a systematic review of prognostic tools for early mortality in hemorrhagic stroke [24]. The authors selected 11 articles (12 prognostic tools), but validation data were reported for only one of the prognostic tools. The Hemphill-intracerebral hemorrhage (ICH) model had the largest number of validation cohorts (nine articles) and showed good performance with a pooled AUC of 0.80 (95%CI 0.77 to 0.85). The second study was a systematic review of prognostic models to predict survival in patients with acute stroke. The authors found 83 models, but only three models were externally validated and showed fair to good discrimination [25]. The final study was a systematic review of prediction models for recurrent stroke and myocardial infarction after stroke. The authors showed that the models for recurrent stroke discriminate poorly between patients with and without a recurrent stroke with the pooled AUCs of 0.60 (95% CI 0.59 to 0.62) for the Essen Stroke Risk Score (ESRS) and 0.62 (95% CI 0.60 to 0.64) for the Stroke Prognosis Instrument II (SPI-II) [26].

Our findings suggest that some of the current prognostic models for predicting complete recovery from ischemic stroke may be clinically useful when applied to patients from high income countries who have experienced moderately severe ischemic stroke. Model No. 9 which was developed by Johnston et al. [14] suggests that the model was not over-fitted to the data set and is likely to be useful in predicting complete recovery from ischemic stroke in a similar population. Models No.3, 6, 13 and 15 involving eight predictors, including NIHSS score, age, infarct volume, history of diabetes mellitus and stroke, prestroke disability, small-vessel stroke and tissue-type plasminogen activator (t-PA use). Some were overlapped among the models as shown in Table 2. These models fulfilled the majority of the methodological requirements and showed acceptable performances in the external validation for both discrimination and calibration. We recommend that these models should be used in other settings.

## Conclusions

This systematic review has shown that, while many prognostic models have been published, they are rarely validated in external populations, and most of the models were developed from elderly patients with moderately severe ischemic stroke, mainly in high income countries. There is a need for the development of models in other settings, especially in low and middle income populations. All models should be validated, and performance measures should be reported which address the two key issues of discrimination and calibration.

## Additional file

Additional file 1: The databases and search strategies use in the systematic review. (PDF 134 kb)

## Abbreviations

AUC: Area under a receiver operating characteristic (ROC) curve
BI: Barthel Index
C-statistics: Concordance statistics
CT: Computerized tomography
ESRS: Essen Stroke Risk Score
GOS: Glasgow Outcome Scale
MRI: Magnetic resonance imaging
mRS: Modified Rankin Scale
NIHSS: National Institutes of Health Stroke Scale
OHS: Oxford Handicap Scale
SPI-II: Stroke Prognosis Instrument II
