# Supplementary material for: Prognostic models for complete recovery in ischemic stroke: a systematic review and meta-analysis
Source: BMC Neurol. 2018 Mar 9;18:26. doi: 10.1186/s12883-018-1032-5 (PMC5845155; doi:10.1186/s12883-018-1032-5)
Supplement: Supplementary file 1 — The databases and search strategies use in the systematic review. (PDF 134 kb) [file 12883_2018_1032_MOESM1_ESM.pdf]

The databases and search strategies use in the systematic review

1. MEDLINE (Pubmed) Retrieved 4 December 2017

#1	((((((“Cerebrovascular Disorder”) OR “ischemic stroke”) OR “Brain ischemia”[MeSHTerms]) OR “cerebral infarction”[MeSHTerms]) OR Stroke[MeSHTerms]) OR “Cerebral thrombosis”) OR “Cerebral embolism”	304,517
#2	(((“barthel index”) OR “modified rankin scale”) OR “Glasgow Outcome Scale”) OR “Oxford handicap scale”	13656
#3	(((((Rehabilitation[MeSHTerms]) OR “Recovery of Function”[MeSHTerms]) OR “functional independence”) OR “Function Recoveries”) OR “Function Recovery”) OR “stroke outcome ”	564,714
#4	(model AND (prognosis OR predict*))	315,862
#5	(“cerebral hemorrhage”[MeSHTerms]) OR “Intracerebral Hemorrhage”	36631
#6	#1 AND #2 AND #3 AND #4	169
#7	#6 NOT #5	156

2. SCOPUS Retrieved 4 December 2017

#1	TITLE-ABS-KEY ((“Cerebrovascular Disorder” OR “ischemic stroke” OR “Brain ischemia” OR “cerebral infarction” OR stroke OR “Cerebral thrombosis” OR “Cerebral embolism”))	449,904
#2	TITLE-ABS-KEY ((“barthel index” OR “modified rankin scale” OR “Glasgow Outcome Scale” OR “Oxford handicap scale”))	17,342
#3	TITLE-ABS-KEY ((rehabilitation OR “Recovery of Function” OR “functional independence” OR “Function Recoveries” OR “Function Recovery” OR “stroke outcome ”))	330,593
#4	TITLE-ABS-KEY ((model) AND (prognosis OR predict*))	1,535,174
#5	TITLE-ABS-KEY ((“cerebral hemorrhage” OR “Intracerebral Hemorrhage” OR “hemorrhagic stroke” OR hemorrhage))	409,799
#6	(#1 AND #2 AND #3 AND #4) AND NOT #5	261

3. MEDLINE (Ovid) Retrieved 4 December 2017

#1	((Cerebrovascular Disorder or ischemic stroke or Brain ischemia or cerebral infarction or Stroke or Cerebral thrombosis or Cerebral embolism) not (cerebral hemorrhage or Intracerebral Hemorrhage or hemorrhagic stroke or hemorrhage)).mp. [mp=title, abstract, full text, caption text]	93659
#2	(barthel index or modified rankin scale or Glasgow Outcome Scale or Oxford handicap scale).mp. [mp = title, abstract, fulltext, captiontext]	7434
#3	(Rehabilitation or Recovery of Function or functional independence or Function Recover# or stroke outcome).mp. [mp = title, abstract, fulltext, captiontext]	65001
#4	(model# and (prognosis or predict*)).mp. [mp = title, abstract, fulltext, captiontext]	95229
#5	1 and 2 and 3 and 4	280

4. CENTRAL (The Cochrane Central Register of Controlled) Retrieved 4 December 2017

#1	“Cerebrovascular Disorder”	69
#2	“ischemic stroke”	5226
#3	MeSH descriptor: [[Brain Ischemia]] explode all trees	2926
#4	MeSH descriptor: [Cerebral Infarction] explode all trees	745
#5	MeSH descriptor: [Stroke] explode all trees	7204
#6	“Cerebral thrombosis”	126
#7	“Cerebral embolism”	92
#8	#1 or #2 or #3 or #4 or #5 or #6 or #7	12221
#9	“barthel index”	1688
#10	“modified rankin scale”	1452
#11	“Oxford handicap scale”	49
#12	“Glasgow Outcome Scale”	721
#13	#9 or #10 or #11 or #12	3440
#14	MeSH descriptor: [Rehabilitation] explode all trees	30099
#15	MeSH descriptor: [Recovery of Function] explode all trees	4558
#16	“functional independence”	1684
#17	“Function Recoveries”	0
#18	“Function Recovery”	452
#19	“stroke outcome ”	207
#20	#14 or #15 or #16 or #17 or #18 or #19	34772
#21	model	81018
#22	prognosis	27308
#23	predict*	76980
#24	#21 and (#22 or #23)	19681
#25	MeSH descriptor: [Cerebral Hemorrhage] explode all trees	853
#26	(#8 and #13 #20 and #24) not #25	56

5. ISI Web of Science Retrieved 4 December 2017

#1	TS= ("Cerebrovascular Disorder" OR "ischemic stroke" OR "Brain ischemia" OR "cerebral infarction" OR Stroke OR "Cerebral thrombosis" OR "Cerebral embolism")	256,473
#2	TS= ("barthel index" OR "modified rankin scale" OR "Glasgow Outcome Scale" OR "Oxford handicap scale")	10745
#3	TS= (Rehabilitation OR "Recovery of Function" OR "functional independence" OR "Function Recoveries" OR "Function Recovery" OR "stroke outcome ")	122,971
#4	TS= (Model AND (prognosis OR predict*))	834,965
#5	TS= ("cerebral hemorrhage" OR "Intracerebral Hemorrhage" OR "hemorrhagic stroke" OR hemorrhage)	110,445
#6	(#1 AND #2 AND #3 AND #4) NOT #5	143
